# The virulence factor *ych*O has a pleiotropic action in an Avian Pathogenic *Escherichia coli* (APEC) strain

**DOI:** 10.1186/s12866-016-0654-2

**Published:** 2016-03-10

**Authors:** Livia Pilatti, Jacqueline Boldrin de Paiva, Thaís Cabrera Galvão Rojas, Janaína Luisa Leite, Rogério Arcuri Conceição, Gerson Nakazato, Wanderley Dias da Silveira

**Affiliations:** Department of Genetics, Evolution and Bioagents, Institute of Biology (P.O.Box: 6109), State University of Campinas – UNICAMP (ZIP Code 13083–970), Campinas, São Paulo Brazil; Department of Microbiology, Center of Biological Sciences, State University of Londrina (UEL), Londrina, Paraná Brazil

**Keywords:** *Escherichia coli*, APEC, *ych*O, Virulence, Pathogenicity

## Abstract

**Background:**

Avian pathogenic *Escherichia coli* strains cause extraintestinal diseases in birds, leading to substantial economic losses to the poultry industry worldwide. Bacteria that invade cells can overcome the host humoral immune response, resulting in a higher pathogenicity potential. Invasins are members of a large family of outer membrane proteins that allow pathogen invasion into host cells by interacting with specific receptors on the cell surface.

**Results:**

An *in silico* analysis of the genome of a septicemic APEC strain (SEPT362) demonstrated the presence of a putative invasin homologous to the *ych*O gene from *E. coli* str. K-12 substr. MG1655. *In vitro* and *in vivo* assays comparing a mutant strain carrying a null mutation of this gene, a complemented strain, and its counterpart wild-type strain showed that *ych*O plays a role in the pathogenicity of APEC strain SEPT362. *In vitro* assays demonstrated that the mutant strain exhibited significant decreases in bacterial adhesiveness and invasiveness in chicken cells and biofilm formation. *In vivo* assay indicated a decrease in pathogenicity of the mutant strain. Moreover, transcriptome analysis demonstrated that the *ych*O deletion affected the expression of 426 genes. Among the altered genes, 93.66 % were downregulated in the mutant, including membrane proteins and metabolism genes.

**Conclusion:**

The results led us to propose that gene *ych*O contributes to the pathogenicity of APEC strain SEPT362 influencing, in a pleiotropic manner, many biological characteristics, such as adhesion and invasion of *in vitro* cultured cells, biofilm formation and motility, which could be due to the possible membrane location of this protein. All of these results suggest that the absence of gene *ych*O would influence the virulence of the APEC strain herein studied.

## Background

*Escherichia coli* strains that cause diseases outside the intestine are known as extraintestinal pathogenic *E. coli* (ExPEC). These strains include human uropathogenic *E. coli* (UPEC), neonatal meningitis *E. coli* (NMEC) and avian pathogenic *E. coli* (APEC) [1–3]. APEC are frequently associated with extraintestinal infections in poultry, leading to respiratory or systemic diseases, which are responsible for large economic losses to the poultry industry worldwide [4, 5]. Colibacillosis is a general term used to describe the large number of existing infections, including septicemia, cellulitis, omphalitis, peritonitis, respiratory tract infections, the egg yolk disease and swollen head syndrome. Colisepticemia, the most severe systemic disease, is characterized by pericarditis, perihepatitis and airsacculitis, and it leads to multiple organ failure and death [6, 7]. In APEC, although several virulence factors such as adhesins, secretion and iron uptake systems, increased serum survival and cytotoxic proteins, have already been identified [1, 6, 8], many others could exist and participate in the pathogenicity process. These currently unknown virulence factors could play major roles in pathogenicity and could be significant for the development of measures for controlling the infectious processes.

Adherence to and invasion of host cells are important steps in the pathogenesis of many bacteria [9]. Bacterial adherence is mediated by adhesins, which recognize receptors on the cell surface. Bacteria that invade host cells possess an important advantage in pathogenicity, overcoming the humoral immune response [10]. A large family of bacterial outer membrane proteins facilitates the entry of the pathogen into host cells by allowing tight adherence to and invasion of the cells. This family of proteins interacts with receptors displayed on the cell surface, triggering signaling cascades to rearrange the host cell cytoskeleton and induce the uptake of bacteria [11, 12]. The first two members of this family (intimin and invasin), although acting differently to promote the invasion of host cells, show significant sequence similarity, especially in the amino terminal region [13, 14]. The first invasin (*inv*) to be described is produced by *Yersinia pseudotuberculosis* and *Y. enterocolitica* [14], and it mediates bacterial entry into eukaryotic cells by high-affinity binding to members of the β_1_ integrin family [12, 15], which are heterodimeric integral membrane proteins that mediate communication between the extracellular environment and the cytoskeleton [16]. The intimins, implicated in attaching and effacing lesions, are produced by enterohemorrhagic (EHEC) and enteropathogenic *E. coli* (EPEC) [13, 17]. In contrast to invasin, the receptor for intimin binding is Tir (Translocated Intimin Receptor), a protein that is secreted into the host cell membrane by the bacterium itself [18]. The intimins and invasins have similar domain structures: an N-terminal signal sequence, a conserved β-barrel domain, and a C-terminal passenger domain (the transported part of the protein) [19]. The β-barrel structure is necessary for the passenger domain, which mediates interactions with host cells, to cross the outer membrane [20].

The *in silico* analysis of recently sequenced genomes of some APEC strains [21–23] enabled the identification of possible new virulence genes that could lead to a better understanding of the infectious process. The *in silico* analysis of the sequenced genome of the APEC strain SEPT362 [22, 24] identified a putative invasin gene, which is homologous to the not yet described *ych*O gene from *E. coli* str. K-12 substr. MG1655 (98 % of identity) and is present in 120 sequenced *E. coli* strains (NCBI). The role of this protein in pathogenicity or biological function of the APEC strains was not previously established, and considering the importance of intimin/invasin-like proteins in other Gram-negative pathogens, this work aimed to test the hypothesis that *ych*O might contribute to the pathogenesis of APEC strain SEPT362. In this work we showed that the *ych*O gene is highly expressed in the lungs and spleen during *in vivo* infection assays by strain SEPT362, what suggests the importance of this gene in *in vivo* colonization of the host. A mutant strain for gene *ych*O was constructed for *in vivo* and *in vitro* comparative analysis with the complemented strain and its counterpart wild-type strain. In this study, we demonstrated, for the first time, that the gene *ych*O is expressed *in vitro* and *in vivo* and is involved in the bacterial capacity for adhesion to and invasion of cultivated cells *in vitro*, motility and biofilm formation and also influences the pathogenicity and the expression of many other genes. These properties are important for pathogenicity *in vivo*, and our results suggest an important role for *ych*O in the pathogenesis of strain SEPT362.

## Results and discussion

### *In silico* characterization of the putative invasin gene *ych*O

The APEC strain SEPT362 was isolated from the liver of a laying hen presenting clinical signs of septicemia [24]. A survey of the genome of strain SEPT362 [GenBank: AOGL00000000.1] revealed a putative invasin homologous to the *ych*O gene from *E. coli* str. K-12 substr. MG1655 (98 % identity). To initiate this work, the re-annotation of this gene using RAST [25] was first performed, which showed a signal peptide sequence of 141 bp that was not present in the GI:449323183 protein annotated in the SEPT362 genome. This peptide was considered to be part of the gene because it is known that this N-terminal signal sequence is an important domain of a typical invasin, predicted to mediate the translocation of the protein from the bacterial cytoplasm through the inner membrane [26]. The *ych*O product alignment also showed that different *E. coli* pathotypes possess a peptide signal on this protein, reinforcing the decision to retain this sequence as part of the gene for the mutant construction. This protein contains an N-terminal signal sequence, a conserved β-domain that forms a transmembrane β-barrel structure, and a C-terminal passenger domain that could be exported to the outside of the cell. These domains together are the main characteristics of the intimin/invasin superfamily. The intimin/invasin-like proteins normally form a longer structure, after the passenger domain, composed of repeated bacterial immunoglobulin-like domains (BID). In some cases, C-type lectin-like domains are present at the C-terminus [27]. These structures were not found in the YchO protein.

### *ych*O expression *in vitro* and *in vivo*

**Fig. 1:**
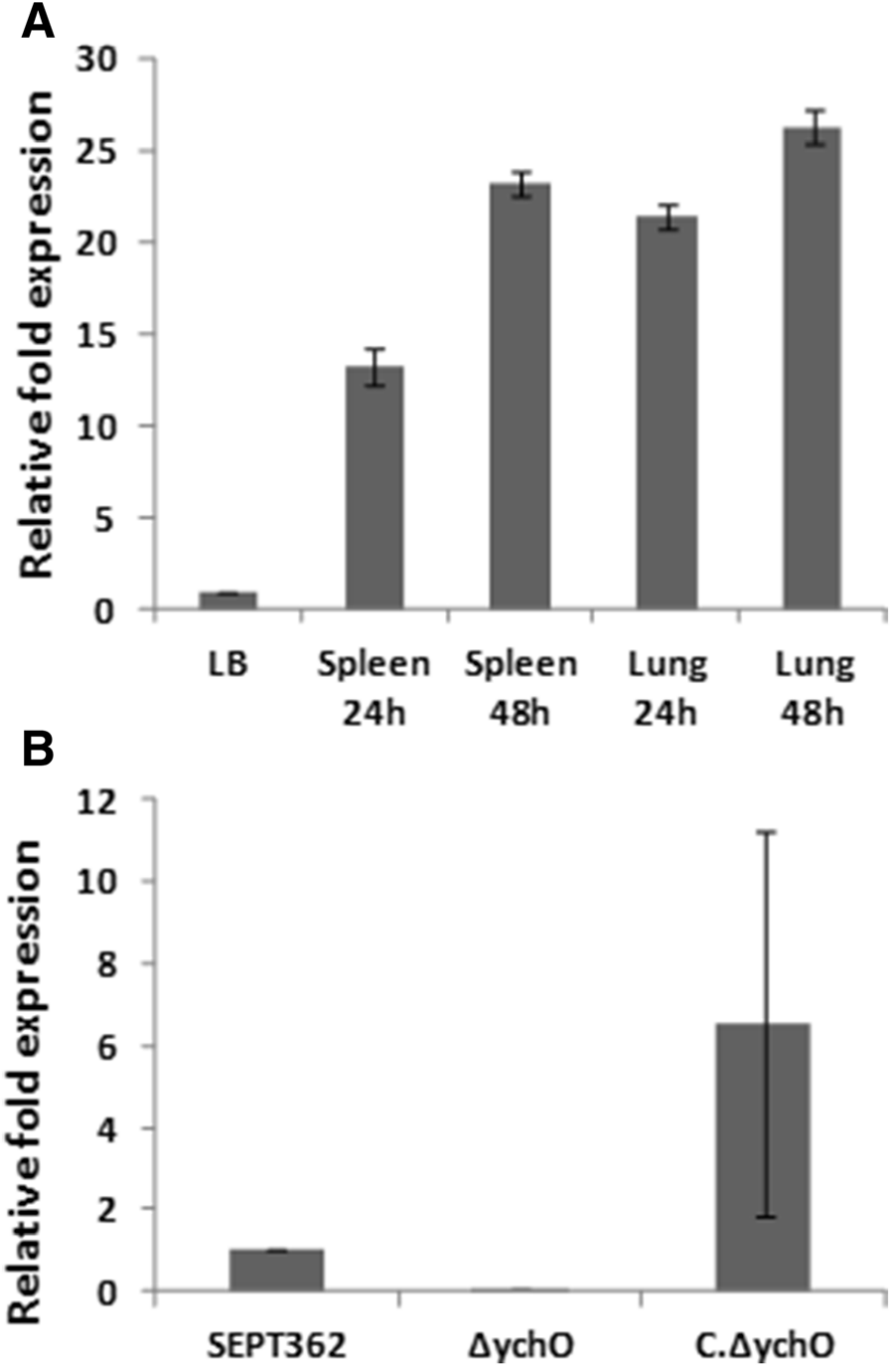
Relative fold expression levels of *ych*O verified using quantitative Real Time-PCR (qRT-PCR). (**a**) Expression of *ych*O in SEPT362 in the spleen and lungs after 24 and 48 h post infection compared to expression *in vitro* using LB media. (**b**) *In vitro* analysis of *ych*O expression in the SEPT362, Δ*ych*O and complemented strains grown in DMEM medium

In this study, first, *ych*O expression was verified in the APEC strain SEPT362 (Fig. 1). The results showed that this gene is expressed not only in culture conditions but is highly expressed in the lungs and spleen of chicks 24 and 48 h after inoculation, which suggests the importance of this gene in *in vivo* colonization of the host. With these results, a mutant strain of the *ych*O gene and a complemented derivative strain containing the wild-type gene on a plasmid were constructed for comparative analysis with the wild-type APEC strain SEPT362. The mutant and complemented strains were verified by quantitative Real Time-PCR (qRT-PCR). The high expression and high standard deviation of *ych*O expression in complemented strain is related to the number of plasmid copies present in this strain, which has an average of 15 copies per cell [28]. A growth curve was constructed for the three strains, using LB and DMEM media. No differences were found between strains in both growing conditions (data not shown).

### The influence of the *ych*O gene on SEPT362 cell adherence

Invasins are a class of proteins that allow bacteria to penetrate cells, normally by tight adherence to and invasion of eukaryotic cells [11, 12]. Adherence and invasion are important steps for bacterial pathogenesis and contribute to the colonization, persistence and dissemination of a pathogen in the host organism [9, 10, 29, 30]. Therefore, understanding the role of a possible invasin might help to elucidate the mechanisms of invasion and pathogenicity of host cells.

**Fig. 2:**
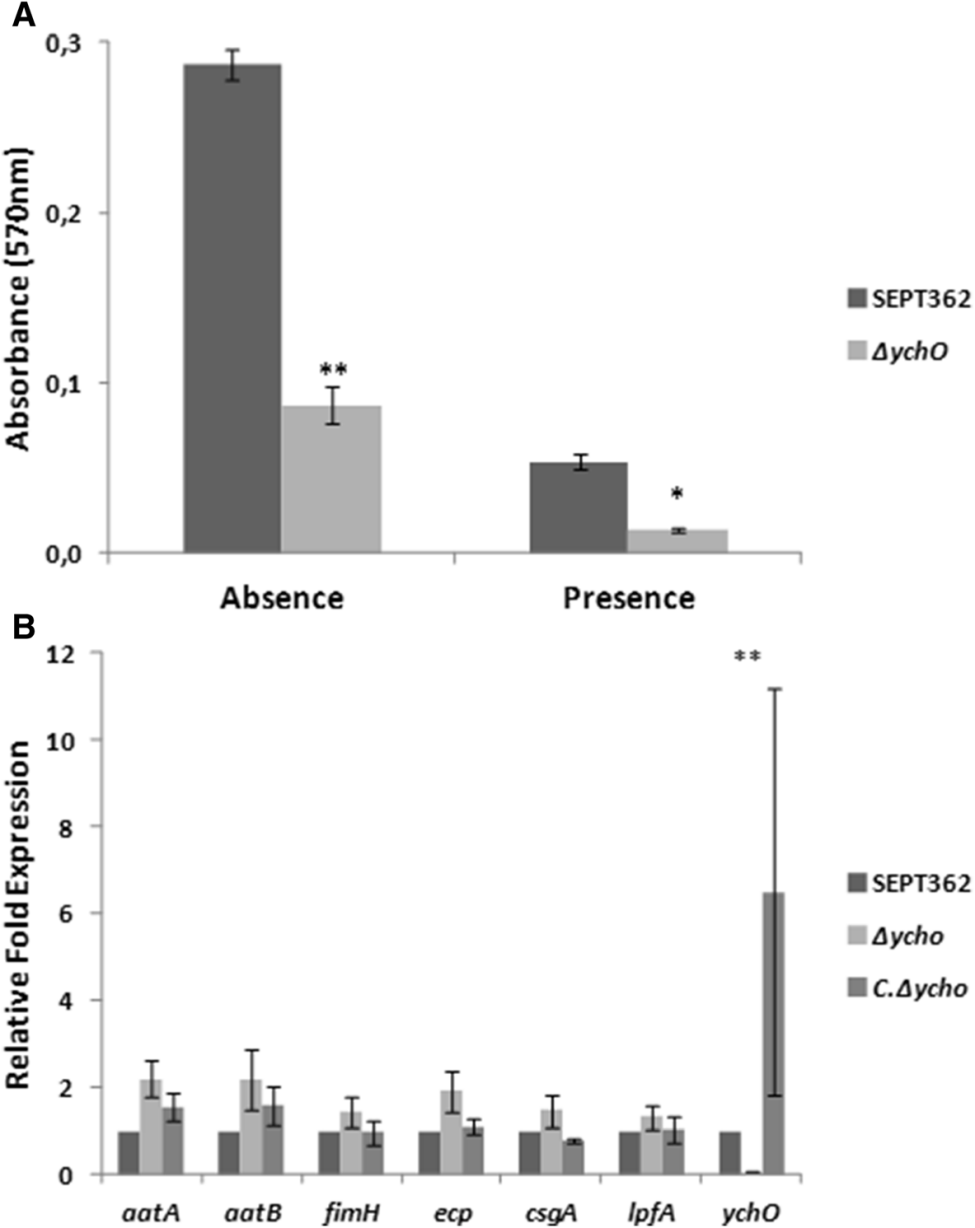
Bacterial adhesion assay. (**a**) Comparison of adhesion between the strains. Quantification of bacteria adhered to the chicken embryo fibroblast cell line (CEF) in the absence or presence of methyl-alpha-D-mannopyranoside. (**b**) Relative fold expression of genes related to adhesion in SEPT362 verified using quantitative Real Time-PCR (qRT-PCR). Statistical significance was determined by Tukey’s test in comparison with SEPT362 (**, p < 0.01; *, p < 0.05)

The capacity of the strains to adhere to chicken embryonic fibroblasts cell line CEF was assessed to understand the possible influence of *ych*O gene on this phenotype. Because type 1 fimbriae binds to D-mannose residues and is important for APEC adherence to chicken cells [31–33], adhesion assays were also performed in the presence of alpha-D-mannopyranoside (D-mannose analog), a potent non metabolized FimH antagonist. The number of bacteria that adhered to pre-fixed CEF cells was significantly lower for the *ych*O mutant than for the wild-type strain in the presence and absence of methyl-alpha-D-mannopyranoside (Fig. 2a). The decrease in the presence of a D-mannose analog shows that the disorder was not due to a disturb in type 1 fimbriae expression. The complemented strain restored the bacterial adhesion. These data suggest that *ych*O is involved in SEPT362 adherence. To investigate whether the change in adhesion ability was due to a disturbance in the expression of other genes possibly related to the adhesion capacity of this strain, different bacterial adhesin genes (adhesin *fim*H [34, 35], *E. coli* common pilus *ecp* [36], long polar fimbria subunit A *lpf*A [37], curlin fimbriae *csg*A [38], autotransporters *aat*A [39, 40] and *aat*B [41]), that were identified by *in silico* analysis to exist in the genome of strain SEPT362, were studied by qRT-PCR (Fig. 2b). The expression levels of these genes were not significantly changed indicating that none of these genes are influenced by the lack of *ych*O, and the decreased adherence of the mutant strain is not due to a decreased expression of those genes. Thus, even without the BIDs and the C-type lectin-like domains [42, 43], the YchO probably acts as an adhesin in APEC strain SEPT362.

### Gene *ych*O contributes to the invasion of chicken embryonic fibroblast cells

**Fig. 3:**
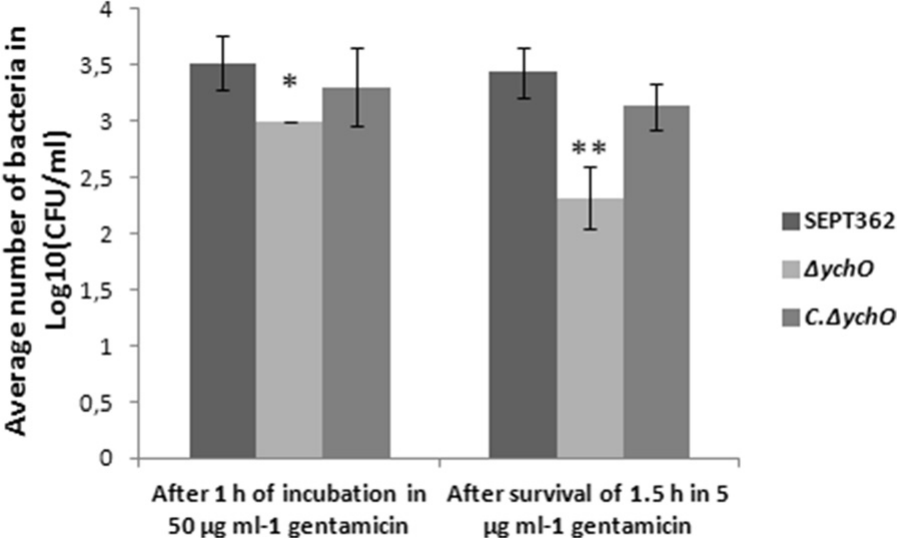
Invasion assay in CEC-32. Comparison of strains after 1 h of incubation in 50 μg ml^−1^ gentamicin and survival after 1.5 h in 5 μg ml^−1^ gentamicin. Statistical significance was determined by Tukey’s test in comparison with the count of the strain SEPT362 (**, p < 0.01, *, p < 0.05)

We assessed the ability of SEPT362 to invade the chicken embryonic fibroblast cell line CEC-32 and the potential contribution of the *ych*O gene to this phenotype. This cell line of avian origin was chosen because it resembles the environment bacteria would find *in vivo*. SEPT362 was able to invade CEC-32 cells efficiently, and the *ych*O gene played an important role in this phenotype. The invasion assay was performed with two sets of cells: one set was lysed immediately after incubation with 50 μg ml^−1^ of gentamycin and the other was lysed after 1.5 h of incubation with 5 μg ml^−1^ of gentamicyn. The number of viable bacteria inside cells after the invasion assay was significantly lower in strain ∆*ych*O than in the wild-type strain, whereas the complemented strain restored this capacity (Fig. 3). The decreased invasiveness was significant, but was not completely abolished. This results suggest that other genes reported to be related to invasion, such as *fli*C (flagellin), *mot*A (flagellar motor protein), *bam*B (outer membrane protein biogenesis), *omp*A (outer membrane protein A), *ibe*B (invasion protein), among others [44–46] that are present in this strain could also contribute to this process. After 1.5 hour of invasion, the number of surviving viable mutants was decreased while the wild-type and complemented strains retained their viability. These data suggest that this protein plays a role not only in invasion, but also in bacterial survival in this cell type.

### Biofilm formation is influenced by *ych*O

**Fig. 4:**
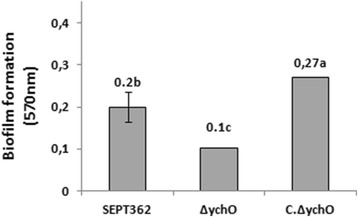
Biofilm formation. Cells were grown for 24 h in polystyrene plates with DMEM. Averages followed by different letters are significantly different (Tukey, p < 0.05)

Biofilms are multicellular communities within a self-produced extracellular matrix attached to a surface [47, 48]. The extracellular matrix helps resist environmental changes and avoid host defenses. Given the capacity of strain SEPT362 to form biofilms on abiotic surfaces [49], we assessed this ability in the mutant strain using the crystal violet biofilm test on polystyrene (Fig. 4). Although the growth curves of strains ∆*ych*O and the C.∆*ych*O did not show any difference when compared with the wild-type strain (data not shown), strain ∆*ych*O formed 50 % less biofilm than the wild-type strain after 24 h, indicating that *ych*O influences biofilm formation. The complemented strain formed 35 % more biofilm than the wild-type strain, which is explained by the overexpression of the gene in its plasmidial structure and suggests the importance of this protein in biofilm formation. These results corroborate a previous report in which a similar putative invasin protein of *Edwardsiella tarda* was found to be essential for biofilm formation [50], even though this protein contains a long structure of repeated BID, whereas *ych*O does not.

### The lack of *ych*O affects the virulence of strain SEPT362

**Fig. 5:**
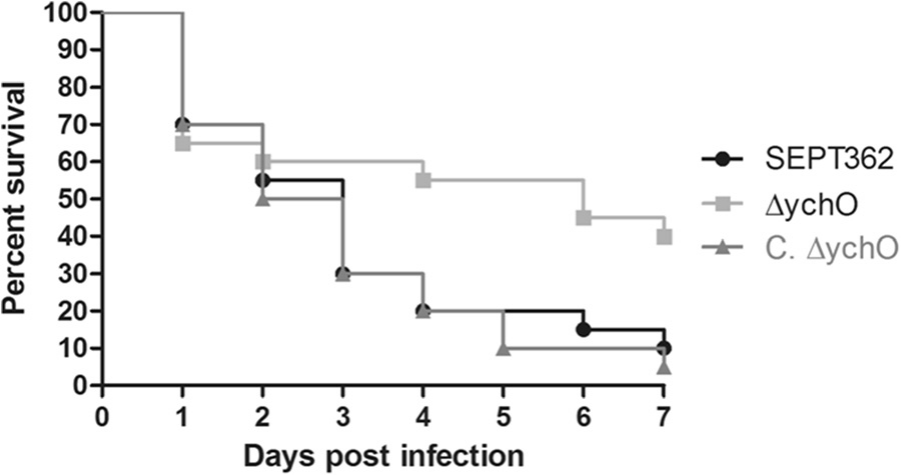
Survival assay. One-day-old broiler chicks were infected with 10^9^ CFU ml^−1^ bacteria (n = 20). There was no statistical significance determined by the Log-rank (Mantel-Cox) test compared with the SEPT362 strain (p > 0.05)

*In vivo* assays were used to evaluate the pathogenicity of strain SEPT362 and its derivatives. For this purpose, separated groups of one-day-old chicks (n = 20 on each group) were infected with the mutant, its complemented derivative and the wild-type strain (Fig. 5). The percent survival of chicks infected with the wild-type was 70 % on the first day, 30 % on the third day, and 10 % at the end of the experiment. For strain ∆*ych*O, the percent survival was 65 % on the first day, 60 % on the third day, and 40 % at the end of the seventh day. No mortality was observed for the negative control infected with 10^9^ CFU ml^−1^ of *E. coli DH10*β (data not shown). Although there were no significant differences among the strains considering the seven days of the experiment (Log-rank test, p > 0.05), a significant change in the survival profile of the mutant strain compared to the wild-type was observed, after the second day (Log-rank test, p > 0.05). The complemented strain presented a profile similar to that of the wild-type strain. The lack of *ych*O gene decreases the virulence of strain SEPT362 after 48 h in the host, which indicates YchO has a role in survival of the strain in the host. Although this gene is also found in non pathogenic *E. coli* strains, all of these results suggest that the absence of gene *ych*O influences the virulence of the APEC strain herein studied in a direct way.

It is known that APEC pathogenesis is controlled by a number of virulence factors, including adhesins (F1-, P-, AC/I-, and F17-fimbriae, curli fimbriae, and afimbrial adhesins), iron acquisition systems (aerobactin and yersiniabactin), hemolysins and the temperature-sensitive hemagglutinin Tsh, antibactericidal factors (outer membrane protein A, a protein for increased serum survival, lipopolysaccharide, K1-capsule, and colicin production), and toxins (heat stable toxin, cyto-/verotoxin, flagella toxin, and vacuolating autotransporter toxin) [4, 51–59]. Many of these virulence factors (*fim*H, *tsh*H, *fli*C, *icm*F, *iuc*C, *yoe*B, among others) have been found in strain SEPT362 by *in silico* search and *in vivo* analysis [22, 24, 49], which most likely makes the pathogenicity of this strain multifactorial. Thus, *ych*O activity is one of several factors that could contribute to SEPT362 pathogenicity, and the full virulence observed in this strain would result from the sum of all the virulence factors present in it.

### The lack of *ych*O affected the expression several genes in SEPT362 strain

**Fig. 6:**
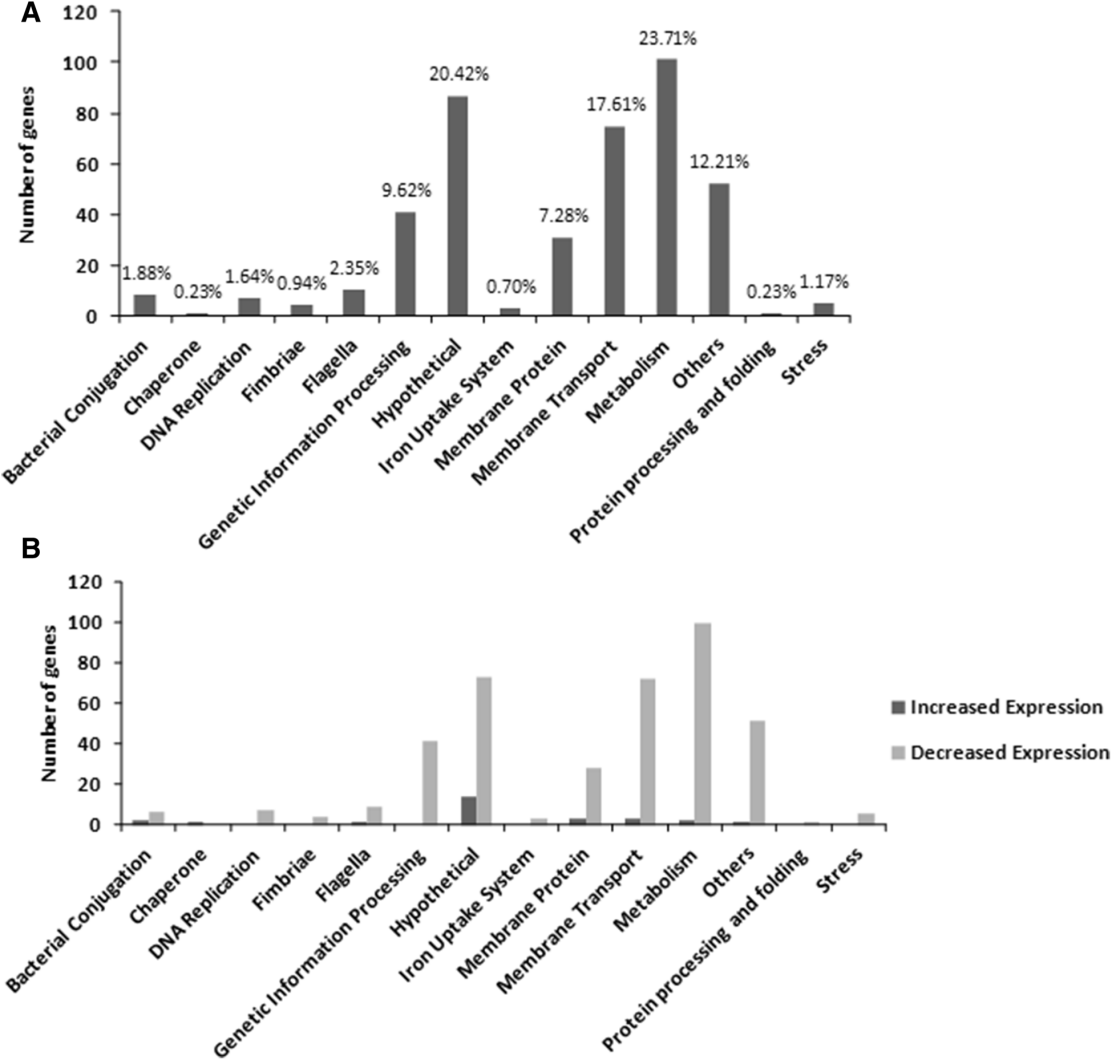
Classification of the genes affected in *ych*O mutant, based on RNAseq analyses. (**a**) Each gene is represented once, and is classified in the most relevant category. Percentage indicates the total number of affected factors in each category, relative to the total number of affected genes. (**b**) Upregulated and Downregulated genes classified in the most relevant category

The results presented so far showed that *ych*O gene has a pleiotropic effect on several biological characteristics, including those related to invasin like proteins, such as biofilm formation, adhesion to and invasion of *in vitro* cultured cells. To further investigate the pleiotropic effect of gene *ych*O to all of these biological characteristics we used the transcriptome sequencing (RNAseq) analysis to compare the transcription profiles of the mutant strain and its wild-type strain. We found that 426 genes were affected upon *ych*O deletion, which were classified into 14 broad categories (Fig. 6a) using Ecocyc and Uniprot. Most of affected genes are classified in metabolism (23.17 %), hypothetical (20.42 %) and membrane transport (17.61 %). Among the altered genes, 93.66 % were downregulated in the mutant (Fig. 6b). The RNAseq was validated by comparison of the expression levels of the genes *csg*A, *lpf*A, *fim*H, *fli*C, *flh*D, *mot*A, *flg*E, *ecp* and *ych*O by qRT-PCR technique. The results were similar for both techniques (data not shown).

The high expression of *ych*O in strain SEPT362 in lungs and spleens during infection and the possible membrane location of protein YchO suggest that the lack of this protein could generate a breakdown of the bacterial membrane, deregulating other membrane-located proteins and membrane transport proteins, triggering a pleiotropic effect, deregulating genes related to metabolism and thus several biological characteristics.

## Conclusions

The adhesion, invasion and biofilm formation results presented in this paper together with the presence of the three basic structures of the protein (peptide signal, β-Barrel and passenger domain), allow us to suggest that gene Ycho protein plays roles as an adhesin/invasion in this specific strain. Despite its short passenger protein structure, without the BID, the protein is important for the virulence of APEC strain SEPT362. With the lack of *ych*O gene, there was a decrease in mortality in chicks. These results, together with the alteration of the expression of 426 genes, led us to propose that *ych*O has a pleiotropic action in APEC strain SEPT362 probably due to an imbalance effect on membrane structure what leads to the altered expression of membrane-related and metabolic genes.

## Methods

### Ethics statement

This study was conducted according to the animal welfare guidelines of the World Organization for Animal Health [60], and approved by the “Ethics Committee on Animal Use-CEUA Unicamp” (Protocol number 2669–1) according to the Brazilian Legislation N° 11794. The chickens were reared in boxes placed in warmed room, according to thermal comfort conditions required by chickens, fed with pathogen-free food and free access to water. After infection, animals were monitored every 6–8 hours. After the experiments, the birds that did not die from colibacillosis caused by inoculated bacteria were euthanized by cervical dislocation and then dissected with aseptic surgical techniques. The birds were sacrificed as a measure for preventing spreading of the disease. All efforts were made to minimize suffering.

### Bacterial strains and growth conditions

The APEC strain SEPT362 (OR:H10) was isolated from the liver of a laying hen presenting clinical signs of septicemia, and it belongs to the bacterial collection of the Bacterial Molecular Biology Laboratory of the Institute of Biology of the State University of Campinas (LBMB) [24, 49]. The genome of SEPT362 has been sequenced [22]. All strains and plasmids used in this study are listed in Table 1. Strains were grown aerobically in Dulbecco’s Modified Eagle’s Medium (DMEM - Nutricell) or Luria-Bertani medium (LB). Antibiotics were added in both media at the following concentrations: 100 μg ml^−1^ ampicillin (Amp), 30 μg ml^−1^ chloramphenicol (Cm), 25 μg ml^−1^ tetracycline (Tet) and 50 μg ml^−1^ kanamycin (Km). Molecular biology techniques were performed as previously described [28].

**Table 1:** Strains and plasmids used in this work

Strain or plasmid	Characteristics	Reference or source
SEPT362	APEC strain isolated from a septicemic laying hen	[24]
SEPT362Cm	SEPT362 with plasmid pKD46Cm	This study
∆*ych*O	*ych*O mutant	This study
C.∆*ych*O	*ych*O mutant complemented with plasmid pC.∆*ych*O	This study
pACYC184	cloning vector	New England Biolabs
pKD4	pANTS derivative plasmid containing FRT-flanked kanamycin resistance	[63]
pKD3	pANTS derivative plasmid containing FRT-flanked chloramphenicol resistance	[63]
pKD46	λ Red recombinase expression plasmid	[63]
pKD46Cm	Plasmid pKD46 with a chloramphenicol cassette insertion	This study
pC.∆*ych*O	Plasmid pACYC184 with *ych*O	This study

### *In silico* characterization of the invasin *ych*O

The NCBI Prokaryotic Genomes Automatic Annotation Pipeline (PGAAP) was employed for gene annotation (http://www.ncbi.nlm.nih.gov/genome/annotation_prok/) of APEC strain SEPT362 [22]. A survey of this genome demonstrated the existence of a gene (*ych*O) described as a putative invasin homologous to the not yet described gene *ych*O from *E. coli* str. K-12 substr. MG1655 and present in contig24 from base 40482 to base 41735. This gene was re-annotated by RAST (*Rapid Annotation using Subsystem Technology*) (http://rast.nmpdr.org/rast.cgi) [25] to confirm the gene size and composition. A topological prediction was obtained by the I–Tasser server [61].

### RNA extraction

Cultures were grown overnight in LB medium at 37 °C, diluted 1:100 in DMEM and grown at 150 rpm to an OD_600_ of 0.5. RNA was extracted by using the RNAeasy Mini Kit (QIAGEN) according to the manufacturer’s protocol.

### *In vivo* analysis of *ych*O gene expression

The expression of the *ych*O gene in the lungs and spleen of infected chicks was verified using qRT-PCR. For this purpose, bacterial cultures were grown overnight in LB medium at 37 °C, washed and resuspended in 0.1 mL of sterile phosphate buffered saline (PBS) at a density of 10^9^ CFU ml^−1^ and injected into the right thoracic air sac of ten one-day-old chicks (Cobb line). At 24 or 48 h post infection, surviving chicks were euthanized, and the lungs and spleens were removed, processed for RNA extraction using the RNAeasy Mini Kit (QIAGEN) and assayed by qRT-PCR.

### Quantitative real time PCR assay

The qRT-PCR assay was performed in a one-step reaction using the ABI StepOne Plus Real-Time PCR (Applied Biosystems), in triplicate (technical replicates) using samples from three independent experiments (biological replicates). Each reaction contained 6 μl of 2X SYBR Green Reaction Mix with Rox, 0.25 μl of SuperScript III RT/Platinum Taq Mix (Invitrogen), 100 nM of each primer, 1 ng μl^−1^ RNA and sufficient DEPC water for a final volume of 12 μl. The *rpo*A gene was used as the endogenous control. Data collection was performed using the ABI StepOne^TM^ Real-Time PCR software v.2.1 (Applied Biosystems). Data were normalized to levels of *rpo*A and analyzed using the comparative critical threshold (*C*_*T*_) method [62]. Error bars represent the standard deviations (SD) of the *C*_*T*_ values.

### Construction of the *ych*O mutant and the complemented strain

The putative invasin gene *ych*O of strain SEPT362 (1,254 bp) was deleted together with the sequence of its signal peptide (141 bp upstream of the *ych*O gene). The mutant strain ∆*ych*O was constructed using the λ Red system [63], with modifications. Briefly, a pair of primers, flanked by 50 nucleotide extensions homologous to the adjacent regions of the target gene, was designed to amplify the kanamycin cassette from the plasmid pKD4. One microgram of purified PCR product was electroporated into strain SEPT362 containing the λ Red recombinase plasmid, pKD46Cm (modified by the insertion of the chloramphenicol cassette amplified from plasmid pKD3). Transformed bacterial cells were plated and grown at 37 °C on LB agar containing kanamycin. Deletion of the *ych*O gene was confirmed by PCR (data not shown) using external primers for the gene. To complement the mutant strain, a DNA fragment covering the *ych*O coding region plus its putative upstream promoter was amplified and cloned into the *Bam*HI and *Sal*I sites of plasmid pACYC184. The plasmid pC.∆*ych*O was transformed into the ∆*ych*O strain, generating the complemented strain C.∆*ych*O. All oligonucleotides used in this work are listed in Table 2.

**Table 2:** Oligonucleotides used for mutant and complement strains construction in this work

Primer	Sequence
Amplification of the Cm cassette	
Forward	GAATTTTTTCGCTATAGTGTAGGCTGGAGCTGCTTC
Reverse	GAATTTTTTCGCTATACATATGAATATCCTCCTTAG
Mutagenesis	
Forward	TATTCTTTAGGGCTATGGTTTTTCATTTTTTACCGGAAGTTACCGACGTTGTGTAGGCTGGAGCTGCTTC
Reverse	AGTCTCGCGTGGAAGCTGCGGTATGGGTGCATCAGGAGCGCATTTTCTGACATATGAATATCCTCCTTAG
Mutagenesis confirmation	
Forward	TACCGGAAGTTACCGACGTT
Reverse	ATCAGGAGCGCATTTTCTGA
Genetic Complementation	
Forward	GCTATAGTCGACGCGAGAAAATACGACAAAAG
Reverse	GTAAGGATCCCACATGCTGAAGAAAATGAA

### Growth curves

Cultures grown overnight in LB medium were adjusted to the same density based on OD_600_, diluted 1:100 and cultivated in DMEM and LB medium at 37 °C with agitation at 150 r.p.m. The OD_600_ was measured every 0.5 h until the bacteria reached the stationary phase.

### *In vivo* pathogenicity experiments

Chick infections were performed as described by de Pace *et al.* [24] with minor modifications. Cultures of wild-type, mutant and complemented strains were grown overnight in LB medium at 37 °C, washed and resuspended in 0.1 mL sterilized PBS at a density of 10^10^ CFU ml^−1^ and injected into the right thoracic air sac of each one-day-old broiler chick. A group of 20 chicks was used for each bacterial culture. The *Escherichia coli* str. K-12 substr. DH10β was used as a negative control. The groups were observed for seven days post infection, and death was recorded every 24 h.

### Bacterial adhesion to Chicken Embryo Fibroblast (CEF) cells

Strains were evaluated for CEF cell adherence in the presence and absence of the D-mannose analog, methyl-alpha-D-mannopyranoside (Sigma cat. n° M6882, Saint Louis, MO, USA), a potent FimH antagonist [64]. *E. coli* adherence to CEF cells was detected by the reduction of the tetrazolium dye MTT to formazan as previously described [65] and by determining the numbers of live bacteria adhering to the surface of pre-fixed cells. CEF cells were cultured in a 96-well plate in DMEM medium supplemented with 10 % fetal bovine serum (FBS) until they reached confluence. The cells were subsequently inoculated with bacteria (OD_600_ ≈ 1.0) at a multiplicity of infection (M.O.I.) of 10:1. The strains were grown with or without 1 % of the mannose analog methyl-alpha-D-mannopyranoside. The *E. coli* HB101 was used as a negative control. The infection time was 1 h, and the multiplication time was 3 h in LB broth. After the multiplication time, cells were washed six times with sterile PBS, and MTT solution [2 mg ml^−1^ (Sigma, cat. n° M2003)] was added for another 2 h of incubation at 37 °C for MTT reduction to formazan. Following the reduction time, the supernatant solution was removed, 100 μl of isopropyl alcohol:hydrochloric acid (24:1) was added, and the absorbance was measured at 570 nm_._

### CEC-32 invasion assay

The chicken embryonic fibroblast cell line CEC-32 [66] was used as a non-phagocytic cell to test APEC-host cell interactions. Fibroblasts were cultivated for 48 h in 24-well culture-plates at 7.5 x 10^4^ cells cm^−2^ in DMEM medium (Nutricell) with 10 % FBS at 37 °C in 5 % CO_2_. Cultures were washed and infected with bacteria at an MOI of 150:1 and incubated for 1 h at 37 °C in 5 % CO_2_. *E. coli* HB101 was used as a negative control. The cells were then washed 3 times with PBS and incubated in DMEM with 10 % FBS and 50 μg ml^−1^ of gentamicin for 1 h at 37 °C in 5 % CO_2_. One set of cells was lysed and plated to count the bacteria. The other set of cultured cells was incubated for 1.5 h in DMEM (Nutricell) with 10 % of fetal calf serum containing 5 μg ml^−1^ of gentamicin before the lysis step. For the lysis step, the wells were washed 3 times with PBS and incubated with 1 ml of 1 % Triton X-100 for 5 minutes. The suspensions were diluted (serial dilutions of 1:10) and plated on LB agar, and the CFU number per ml was determined by counting the colonies for each dilution.

### Biofilm formation

Biofilm formation was analyzed using crystal violet staining as previously described by Christensen *et al*. [67]. Overnight cultures of SEPT362, ∆*ych*O and C.∆*ych*O were inoculated in triplicate into DMEM at a 1:100 dilution in 24-well cell culture plates (polystyrene) to a final volume of 1 ml. The plates were incubated at 37 °C in a 5 % CO_2_ atmosphere for 24 h. The wells were then washed 3 times with 1x PBS, pH 7.4. The cells were fixed with 1 ml of 75 % ethanol, washed 3 more times with 1x PBS and stained with 0.5 % crystal violet for 5 min. After washing 4 times with 1x PBS, pH 7.4, the crystal violet was solubilized by adding 1 ml of 95 % ethanol to each well for 2 minutes. The absorbance of this solution was determined in a spectrophotometer at 570 nm.

### Global gene expression analyses by transcriptome sequencing (RNAseq)

The mRNA transcriptome was isolated using the Ribominus™ Transcriptome Isolation Kit (Yeast and Bacteria) (Invitrogen - Life Technologies) according to the manufacturer’s protocol. The transcriptome sequencing (RNAseq) was performed using the platform HISEQ2000 (ILLUMINA). Samples containing 2 mg of mRNA were used for preparation of cDNA libraries specific sizes that were fragmented enzymatically repaired and linked to adapters discerned in the samples bioinformatics analyzes. After preparation, the libraries were deposited in one slide (flowcell) containing 8 channels (lanes) via the robotic instrument cBot (Illumina). The flowcell was then placed in HiSeq2000 where the sequencing occurred. At the end of the race, bioinformatics analyses were performed for gene expression comparison. The reads were aligned with the Open Read Frames (ORFs) and non-coding transcripts from SEPT362 strain. The alignment was performed by using the Bowtie program [68], allowing 2 mismatches. The transcript expression calculation was based on the number of aligned reads, according to Mortazavi *et al*. (2008) [69]. Only reads with one alignment against the ORFs were considered. Differently expressed ORFs were indentified based on the number of reads with one alignment each. Statistic analyses were performed using R De-Seq [70]. According to the program authors, ORFs less expressed in each of the analyses were excluded from the statistics tests. The validation was performed by qRT-PCR for genes *csg*A, *lpf*A, *fim*H, *fli*C, *flh*D, *mot*A, *flg*E, *ecp* and *ych*O.

### Statistical analysis

All *in vitro* assays were performed in triplicate. The results of adhesion, invasion, biofilm formation and qRT-PCR assays were compared using a Tukey test. Statistical analyses were performed with ASSISTAT Version 7.6 Beta (2012). RNAseq statistic analyses were performed using R De-Seq. For the chick infection assays, the Log-rank (Mantel-Cox) test was performed using GraphPad Prism version 5.00 for Windows, GraphPad Software, San Diego California USA, www.graphpad.com. Differences were considered significant at a P value of <0.05.
